# Effects of Maternal Factors and Postpartum Environment on Early Colonization of Intestinal Microbiota in Piglets

**DOI:** 10.3389/fvets.2022.815944

**Published:** 2022-04-07

**Authors:** Yongshi Li, Yadan Liu, Yijia Ma, Xusheng Ge, Xiaona Zhang, Chunbo Cai, Yang Yang, Chang Lu, Guoming Liang, Xiaohong Guo, Guoqing Cao, Bugao Li, Pengfei Gao

**Affiliations:** ^1^College of Animal Science, Shanxi Agricultural University, Taigu, China; ^2^Inner Mongolia Mengniu Dairy Company Limited, Helingeer Hohhot, China; ^3^Allwegene Technologies Incorporation, Beijing, China

**Keywords:** 16S rRNA, piglets, characteristics of microbial colonization, source of intestinal microbiota, microbial diversity

## Abstract

Intestinal microbiota significantly influences the intake, storage, and utilization of body nutrients, as well as animal growth and development. The establishment of microbiota is affected by many factors, such as delivery and feeding modes, antibiotics, disease, and the surrounding environment. In this study, we selected Chinese indigenous Mashen and Jinfen White pigs as the study subjects. To explore the source and factors affecting the piglet intestinal microbiota, 16S rRNA gene sequencing was performed to analyze the microbial composition of the feces, saliva, vaginal secretions, and colostrum of parturient sows, feces and saliva of newborn piglets, and surrounding environment samples. The results showed that the microbiota of the saliva of sows and piglets is structurally similar to that of the environment and is dominated by the phylum Proteobacteria, including *Acinetobacter, Actinomyces*, and *Pseudomonas*. The core genus in the vaginal secretions and colostrum of sows was *Pseudomonas*. Among the fecal samples, the core bacterial genera in sows before and after delivery were *Clostridium sensu_stricto_1* and *Christensenellaceae_R-7_group*, while in piglets at 1 d of age, *Pseudomonas* and *Escherichia-Shigella* were most abundant. These results indicate that microbiota in feces, colostrum, and vaginal secretions of sows more easily colonized piglet intestines through a symbiotic effect. The environmental and salivary microbiota could also affect the early colonization and succession of the intestinal microbiota of piglets to some extent. This study provides a theoretical basis for sow delivery protection and early nursing of piglets and background for the research and development of microbial agents to improve piglet intestinal health.

## Introduction

The intestines of mammals contain trillions of microbiota, forming a complex ecosystem (1). Intestinal microbiota play an important role in improving host immunity, digestion, metabolism, intestinal endocrine function regulation, and endotoxin removal (2, 3). For instance, stimulating intestinal microbiota can induce immune system maturation, thus protecting the host from infection, maintaining intestinal homeostasis, and promoting livestock and poultry health (4). Furthermore, contact between the body and microbiota early in life affects immune system formation; symbiotic bacteria colonization contributes to the development, expansion, and conditioning of the mucosal immune system, thus directly or indirectly affecting immune system maturation (5). A study on the intestinal development of germ-free and specific pathogen-free mice revealed that the failure of microbial colonization during the critical period of infancy or a lack of contact with the microbiota could cause potential and irreversible harm to the host, resulting in incomplete immune system development in adulthood (6). It has been found that *Lactobacillus rhamnosus* GG increased the daily gain and total weight of piglets, reduced the rate and duration of diarrhea, and improved the biological, physical, and immunologic barriers of intestinal mucosa of piglets (7). Additionally, exposure to microbiota at an early age helps prime the immune system and reduces the susceptibility to autoimmune diseases (8).

Early intestinal microbial colonization is essential for piglet health, and it is generally believed that piglets are in germ-free conditions before birth. Several studies have shown that although the maternal uterus is sterile, during delivery, the fetal membrane ruptures, and the fetus comes into contact with the microbiota in the birth canal, leading to the colonization of some microbiota in the fetus (9–12). The maternal birth canal, saliva, breast milk, farrowing bed environment, and feed are the primary means by which piglets acquire microbiota (13). Studies have shown that vaginally delivered babies have microbiota similar to the vaginal microbiota of the mother, mainly *Lactobacillus* and *Prevotella* (14). After parturition, piglets can obtain energy sources and improve their immunity through breastfeeding; simultaneously, colonization of the intestine with microbiota from breastmilk is promoted (15, 16). The farrowing environment, including the feed and stall, is an important factor affecting intestinal microbial colonization in piglets (17, 18). The farrowing beds inhabited by newborn piglets are associated with microbiota from sow feces, saliva, and other sources. Accordingly, it has been reported that suckling piglets consume ~20 g of maternal feces on average daily (19). Coprophagy may also provide nutrients, gradually establishing core intestinal microbiota (20).

The early postnatal period is a critical stage of pig development as the new pig transitions from a sterile intrauterine environment to an external environment full of antigens and pathogenic challenges. At this period, the piglets are susceptible to external factors due to incomplete body development, imperfect digestion, absorption, and body immunity, which is easy to cause diarrhea and other diseases (21). The intestinal tract is not only the main site for the absorption and metabolism of animal nutrients but also the largest immune organ of the body (22); therefore, early colonization by intestinal microbiota has great significance for the growth and development of piglets. At present, several studies have focused on the source of intestinal microbes in piglets but there is no definite conclusion. THROUGH 16S rRNA gene sequencing, a prior study found that microbiota from the maternal and surrounding environments may play an important role in the microbial succession of newborn piglets after birth (13). Another research has shown that maternal milk and fecal microbiota may be involved in the establishment of intestinal immune and barrier functions in newborn piglets (23). Furthermore, the difference in the microbial colonization between local and hybrid pig breeds has not been clarified to data.

Mashen pigs are an outstanding indigenous breed native in Shanxi Province characterized by high fertility, good meat quality, strong resistance to stress, and a slow growth rate (24, 25). On the other hand, Jinfen White pigs are a local hybrid breed of multiple pigs, including Mashen (6.25%), Taihu (3.13%), Landrace (40.62%), and Yorkshire pigs (50%), with a significantly higher growth rate than that of Mashen pigs (26). Therefore, in this study, Jinfen White and Mashen pigs were used as experimental models, and 16S rRNA gene sequencing was used to analyze the microbial structure of piglet feces and saliva, vaginal secretions, colostrum, feces, and saliva of parturient sows, as well as the surrounding environment, to explore the source and factors influencing the intestinal microbiota of piglets, and to provide a theoretical reference for studies on early growth and development of piglets.

## Materials and Methods

### Animals and Sample Collection

All procedures involving animals were conducted according to the guidelines for the care and use of experimental animals established by the Ministry of Agriculture of China. The Animal Care and Use Committee of Shanxi Agricultural University approved this study.

The experimental animals used in this study were raised at Datong pig breeding farm which is located in north China (113°27 ′E, 40°04′ N). Piggery is a light steel structure, double slope roof, color steel plate covered with meters long and 18 meters wide, and cool cell on one end and sides and fans on the other end. The project was approved by the Chinese Agricultural Industry Standards committee (NY/T 65-2004). The diets were adapted for the nutritional requirements of sows for the gestation and lactation period. In general, pregnant sows were fed twice a day and were given free water. In the month before birth, sow feeding increased by 20%. Lactating sows were fed 3 to 4 times a day. Piglets stayed with their respective mother until weaning at the age of 28 days. The piglets were fed with trough feed 7 days after birth, weaned on day 28, transferred to the nursing house after weaning, and entered the fattening stage at 70 d of age. The feed formulation of sows and piglets is provided in Supplementary Table 1.

To evaluate the effects of maternal factors and the postpartum environment on early intestinal microbial colonization in piglets, the feeding experiment was carried out in July 2019, where the average temperature was between 19 and 21.8°C and the average precipitation was 246.9 mm. Three sows (24 months of age) were selected, respectively, from the Mashen and Jinfen White breeds for synchronized estrus and mating. On average, the number of piglets born alive was 12.1 ± 0.8 (mean ± SEM) for Mashen sows and 11.3 ± 0.7 for Jinfen White sows. And the birth weight of Mashen piglets and Jinfen White piglets were 1.26 ± 0.05 kg and 1.44 ± 0.04 kg, respectively. The sows had no adverse symptoms before and after delivery, and none of the diets contained antibiotics or other drugs.

During parturition of the sow, vaginal secretions were collected with sterile cotton swabs and placed in sterile containers. One milliliter of colostrum was manually collected immediately following alcohol swabbing of teats from all sows after sow parturition. Additionally, fecal samples were collected from the rectum of sows with sterile swabs and enriched in the same conditions at 5 and 3 d before and on the day of parturition (1 d), and 4 and 7 d postpartum. Furthermore, saliva samples from the oral cavity of sows were collected using sterilized cotton swabs 3 d before parturition, on the day of parturition (1 d), and 4 d postpartum. Three piglets were selected from the same litter and fecal samples were collected at 1, 4, and 7 days of age, respectively. Saliva samples from the oral cavity of piglets were collected using sterilized cotton swabs at 1 and 4 d after birth. Environmental microbiota on the floor surface of the farrowing bed were collected using sterilized cotton swabs. To prevent contamination, separate rubber gloves and sterile cotton swabs were used for the collection of each sample, and all samples were frozen in liquid nitrogen and stored at −80 °C until further analysis.

### DNA Extraction and Sequencing

Microbial genomic DNA was extracted from the samples using the QIAamp Fast DNA Stool Mini Kit (Qiagen, Valencia, CA, USA) (27). Primers were specific for the V3 and V4 regions of the bacterial 16SrRNA gene in the NCBI database (28), and 341F (5′-CCTAYGGGRBGCASCAG-3′) and 806R (5′-GGACTACNNGGGTATCTAAT-3′) were amplified using PCR. The amplification program consisted of 1 pre-denaturation cycle at 95°C for 3 min, followed by 27 cycles of denaturation at 95°C for 30 s, annealing at 55°C for 30 s, extension at 72°C for 30 s, and finally, 1 extension cycle at 72°C for 10 min. Thereafter, PCR products were recovered using a DNA Gel Recovery Kit (Axygen Inc, Corning, NY, USA), purified using a DNA gel extraction kit (Tiangen, Beijing, China), and sequenced on an Illumina MiSeq sequencing platform (Illumina, San Diego, CA, USA) (29, 30). Sequences with more than 10% of unknown nucleotides (N) in the raw data and sequence quality lower than 80% (*Q* > 20) were removed. and the sequences were spliced using FLASH software; the minimum overlap length was 10 bp, and a maximum mismatch rate of 0.2 was allowed in the overlap region (31). Subsequently, quality control and data optimization of the splice quality were performed using Trimmomatic software.

### Bioinformatics Analysis

Using UPARSE version 7.1 to cluster the Operational taxonomic units (OTUs) with 97% similarity cutoff (32, 33), chimeric sequences were identified and removed. The taxonomy of each OTU representative sequence was analyzed by RDP Classifier version 2.2 (34) against the Silva 16S rRNA database (e.g., Silva v138) using confidence threshold of 0.7. Mothur software (version v.1.30.1) (35) was used to calculate alpha diversity index under different random sampling, a dilution curve plot was constructed using tools in R studio (v.3.0.2) to reflect the sampling sufficiency (36). For alpha diversity analysis, ACE, Shannon, Coverage, and PD indices were used to determine microbial community richness, microbial community diversity, microbial community coverage, and microbial phylogenetic diversity, respectively. Furthermore, beta diversity among samples was calculated based on unweighted UniFrac distance and principal coordinate analysis (PCoA) (37). Heatmaps of different pathways were generated using edgeR in R (38, 39).

### Data Availability

The 16S rRNA gene sequence data used in this study were deposited in the NCBI SRA database under the accession number SRP321842.

### Statistical Analysis

Diversity indices were analyzed using one-way ANOVA, and multiple comparisons were conducted using the Tukey. Data were presented as mean ± standard deviation and analyzed using GraphPad Prism 7.0 (GraphPad Software, San Diego, CA, USA). Statistical significance was set at *P* < 0.05. Difference analysis of microbiota in different samples based on the community abundance data, using one-way ANOVA and Kruskal-Wallis H test, the hypothesis test was carried out on the species of microbial communities of different samples, the significant level of species abundance difference was evaluated, and the species of significant difference between samples was obtained. Statistical significance was set at *P* < 0.05 and *P* < 0.01.

## Results

### Quality Control of the 16SrRNA Gene

**Figure 1:**
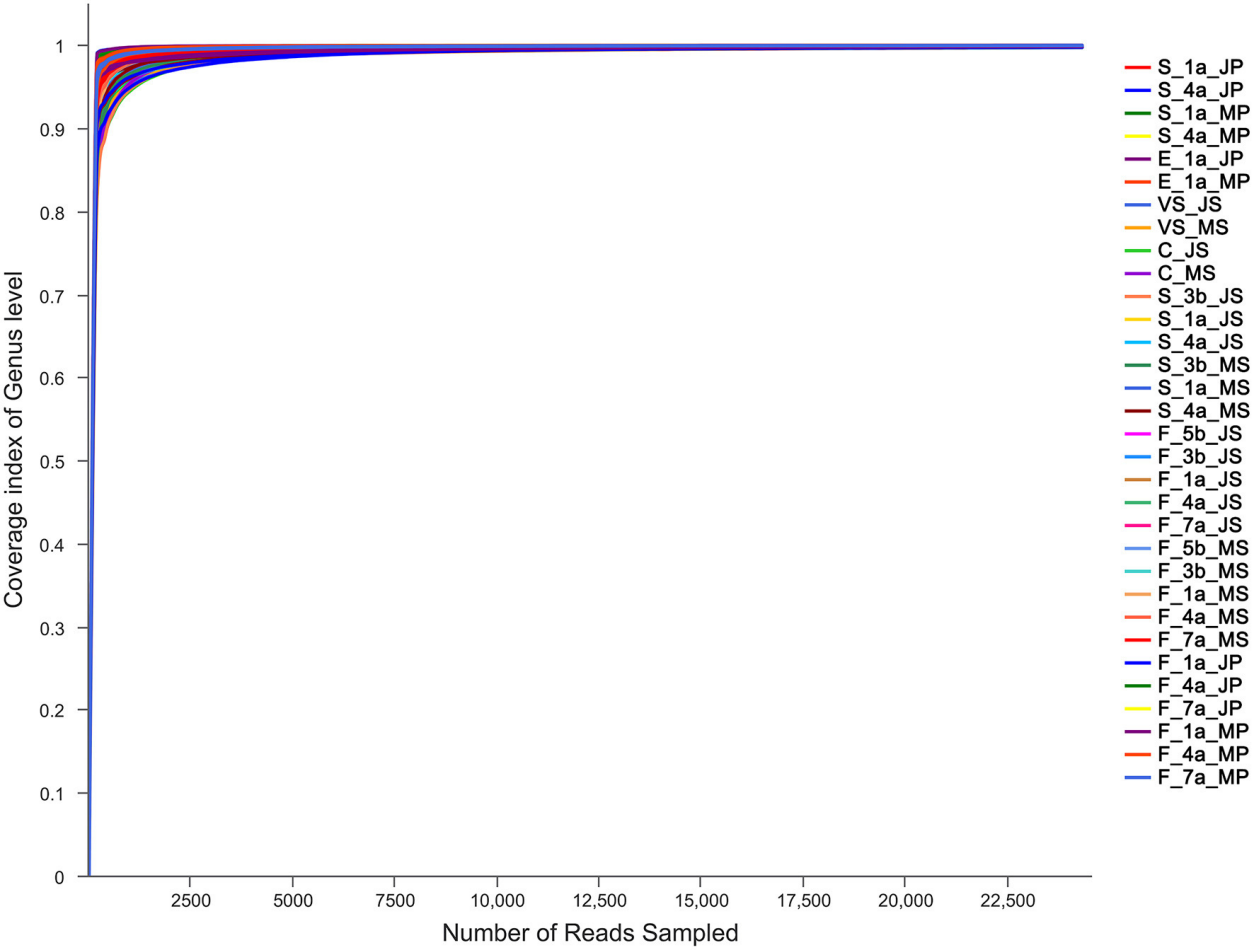
Results of sequencing coverage of Mashen pig and Jinfen White pig. S, indicates the saliva samples; C, indicates the colostrum samples; F, indicates the fecal samples; VS, indicates the vaginal secretions; E, indicates the farrowing bed environment; JS, indicates Jinfen White sows; JP, indicates Jinfen White piglets; MS, indicates Mashen sows; MP, indicates Mashen piglets; 5 b indicates the samples collected at 5 d before sow parturition; 3 b indicates the samples collected at 3 d before sow parturition; 1 a indicates the samples collected at 1 d after piglet birth or the day of sow parturition; 4 a indicates the samples collected at 4 d after piglet birth or sow parturition; 7 a indicates the samples collected at 7 d after piglet birth or sow parturition.

To ensure sample sequencing homogeneity, raw data were flattened according to the minimum number of sample sequences. The microbiota in the sequenced samples were mainly distributed into 38 phyla, 67 classes, 148 orders, 269 families, and 768 genera (Supplementary Table 2). A total of 5,053,253 sequences with an average length of 438 bp were obtained (Supplementary Table 3). With an average coverage of the samples >99%, the curve tended to be flat, eventually reaching a plateau, indicating that the amount of sequencing data was reasonable and can be utilized to obtain relevant information on microbiota diversity in the sample (Figure 1).

### Microbial Diversity Analysis of Different Samples

Shannon, ACE, and PD indices were used to comprehensively evaluate the alpha diversity of microbiota in different samples from sows and piglets of the two breeds (Table 1). The microbial community diversity in saliva before parturition was significantly higher than that after parturition (*P* < 0.05) in Mashen sows. Contrastingly, it was lower at parturition and increased after parturition in Jinfen White sows, however, the difference was not significant (*P* > 0.05). The microbial community richness in the saliva of the two sow breeds was lowest during parturition, increasing significantly after parturition, and subsequently returning to prepartum levels (*P* < 0.05). The microbial phylogenetic diversity of microbiota in sow saliva was the lowest on the day of parturition and then increased gradually. The microbial Shannon index in the saliva of the two piglet breeds was lower on the 1st day after birth and then increased significantly after 4 days (*P* < 0.05). In contrast, ACE and PD indices were higher at birth, indicating that piglet microbial community richness and phylogenetic diversity were higher at birth and then decreased significantly (*P* < 0.05). Although the microbial community diversity in the feces of the two sow breeds was higher before and during parturition, it did not significantly differ from that after parturition (*P* > 0.05). Similarly, microbial phylogenetic diversity was highest 5 days before parturition then decreased later, but the overall difference was insignificant (*P* > 0.05). The microbial community richness in the feces of the two sow breeds was lowest during parturition, then increased insignificantly after parturition (*P* > 0.05). On the one hand, the community diversity of microbiota in the feces of Mashen piglets increased significantly with increasing age, while the ACE and PD indices decreased at first and then increased. On the other hand, the fecal microbial Shannon, ACE, and PD indices of Jinfen White piglets decreased significantly after birth (*P* > 0.05) and then increased.

**Table 1:** Sample microbial diversity index table of Jinfen White pig and Mashen pig.

**Animal**		**Species**	**Time**	**Shannon**	**ACE**	**PD**
Sow	Mashen pig	Saliva sample	3 d before parturition	3.53 ± 0.41^a^	284.96 ± 18.35^b^	28.47 ± 0.59
			On the day of parturition	2.57 ± 0.22^b^	279.59 ± 18.02^b^	24.09 ± 1.57
			4 d after parturition	2.52 ± 0.19^b^	331.29 ± 23.71^a^	27.82 ± 3.33
		Colostrum	At the day of parturition	2.87 ± 0.47	210.82 ± 10.43	27.28 ± 0.76
		Vaginal secretion	At the day of parturition	3.62 ± 0.35	373.84 ± 66.69	35.45 ± 2.77
		Fecal sample	5 d before parturition	3.26 ± 0.25	167.42 ± 12.60	20.25 ± 2.77
			3 d before parturition	3.33 ± 0.12	169.5 ± 18.10	18.29 ± 2.09
			On the day of parturition	3.25 ± 0.18	163.41 ± 22.98	16.36 ± 1.74
			4 d after parturition	2.98 ± 0.23	175.62 ± 8.07	18.39 ± 0.46
			7 d after parturition	3.13 ± 0.19	161.82 ± 25.23	17.27 ± 1.91
	Jinfen White pig	Saliva sample	3 d before parturition	2.80 ± 1.23	307.76 ± 24.69^a^	30.53 ± 3.10^a^
			On the day of parturition	2.42 ± 0.67	246.55 ± 35.89^b^	22.05 ± 2.03^c^
			4 d after parturition	2.65 ± 0.26	320.22 ± 39.15^a^	28.36 ± 2.37^b^
		Colostrum	At the day of parturition	3.33 ± 0.25	276.17 ± 52.87	32.68 ± 3.92
		Vaginal secretion	At the day of parturition	2.80 ± 1.18	300.24 ± 31.76	31.25 ± 5.88
		Fecal sample	5 d before parturition	3.13 ± 0.16	161.07 ± 6.89^b^	18.74 ± 1.92
			3 d before parturition	3.13 ± 0.2	164.47 ± 13.08^ab^	18.00 ± 1.61
			On the day of parturition	3.30 ± 0.01	153.35 ± 12.2^ab^	18.12 ± 1.85
			4 d after parturition	2.92 ± 0.39	166.87 ± 13.53^ab^	17.21 ± 0.45
			7 d after parturition	3.09 ± 0.14	181.82 ± 12.38^a^	18.40 ± 1.15
Piglet	Mashen pig	Saliva sample	On the day of parturition	1.52 ± 0.35^b^	294.03 ± 12.06^a^	24.35 ± 1.05^a^
			4 d after parturition	2.81 ± 0.11^a^	184.98 ± 55.63^b^	14.22 ± 1.88^b^
		Environment	On the day of parturition	2.57 ± 0.26	394.38 ± 31.68	30.68 ± 1.77
		Fecal sample	On the day of parturition	1.54 ± 0.88^b^	180.94 ± 113.12^a^	15.73 ± 9.15
			4 d after parturition	2.20 ± 0.52^a^	92.58 ± 17.62^b^	9.73 ± 2.27
			7 d after parturition	2.25 ± 0.15^a^	115.27 ± 19.25^ab^	11.67 ± 0.23
	Jinfen White pig	Saliva sample	On the day of parturition	0.85 ± 0.36^b^	235.71 ± 35.12^a^	21.30 ± 9.48^a^
			4 d after parturition	2.62 ± 0.22^a^	165.01 ± 38.48^b^	13.95 ± 4.55^b^
		Environment	On the day of parturition	2.43 ± 0.06	346.66 ± 56.05	28.93 ± 2.02
		Fecal sample	On the day of parturition	2.08 ± 0.55^a^	265.99 ± 154.87^a^	25.88 ± 16.48^a^
			4 d after parturition	1.19 ± 0.38^b^	87.54 ± 8.66 ^c^	7.95 ± 1.14^b^
			7 d after parturition	2.23 ± 0.44^a^	108.21 ± 7.50^b^	10.79 ± 1.9^b^

*Values are represented as mean ± SD, and the variant letter in the same column indicates significant difference when P < 0.05*.

### Sample Principal Coordinate Analysis

**Figure 2:**
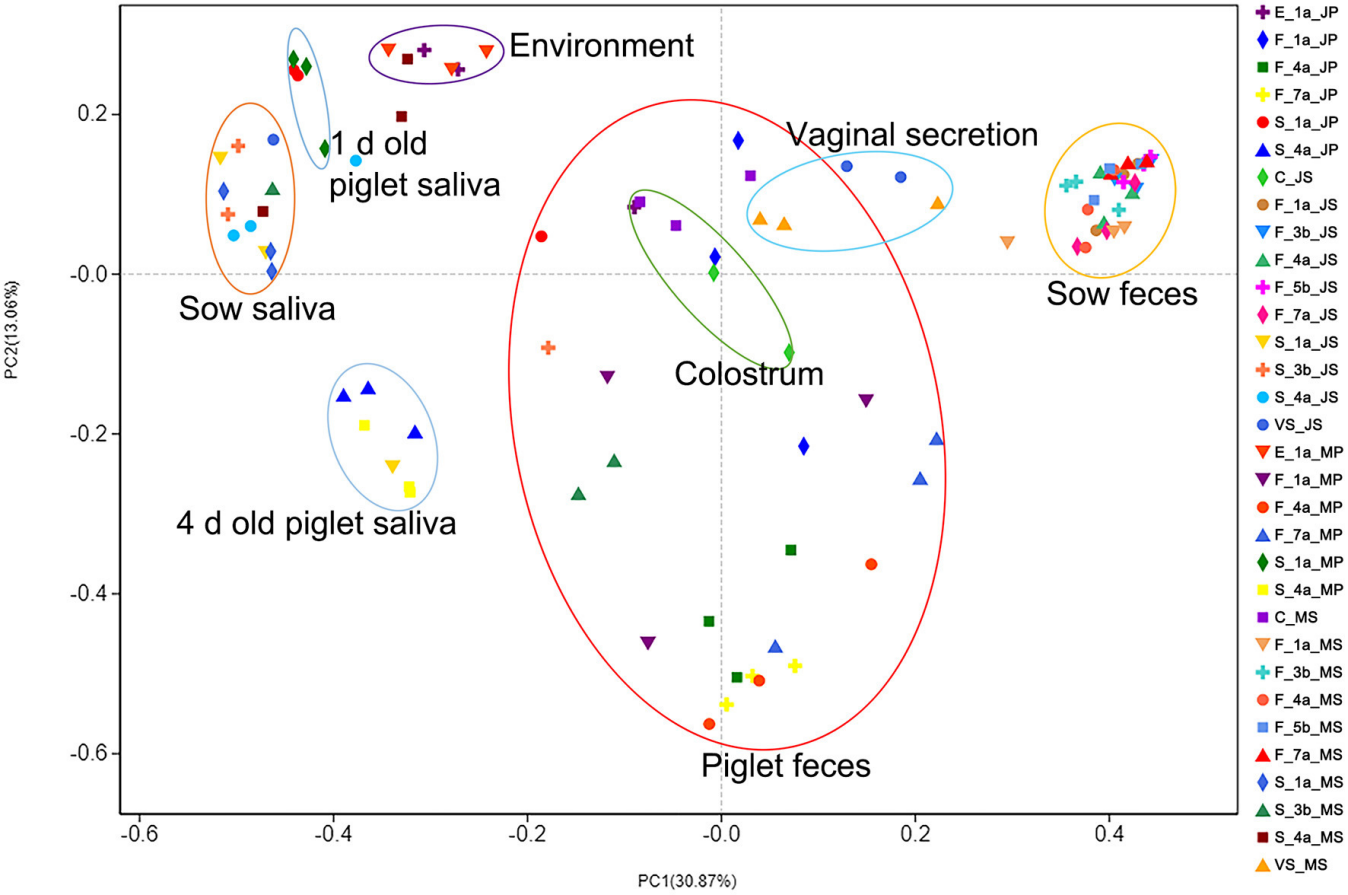
PCoA analysis results of microbial diversity of samples from Jinfen White pig and Mashen pig. Principal coordinate analysis (PCoA) results of the sow and piglet saliva, farrowing bed environment, sow and piglet feces, colostrum, and sow vaginal secretion. Principal coordinate 1 (PC1) represents microbiota abundance, principal coordinate 2 (PC2) represents microbial community evenness.

In the sample principal coordinate analysis (PCoA) results, principal coordinate 1 (PC1) represented microbiota abundance, accounting for 30.87% of the total variation, including, from left to right, sow and piglet saliva, farrowing bed environment, piglet feces, colostrum, sow vaginal secretion, and sow feces. Principal coordinate 2 (PC2) represented microbial community evenness, accounting for 13.06% of the total variation (Figure 2). The fecal microbiota of the two sow breeds clustered closely and significantly differed from those of the other samples, indicating that the intestinal microbiota of adult pigs significantly differed from that of piglets. The fecal microbiota of the two piglet breeds was dispersed between the sows and environmental samples, indicating that piglet intestinal microbiota was unstable and was easily affected by various factors.

### Relationship Between Early Microbial Colonization and Maternal Factors and Environment in Piglets

**Figure 3:**
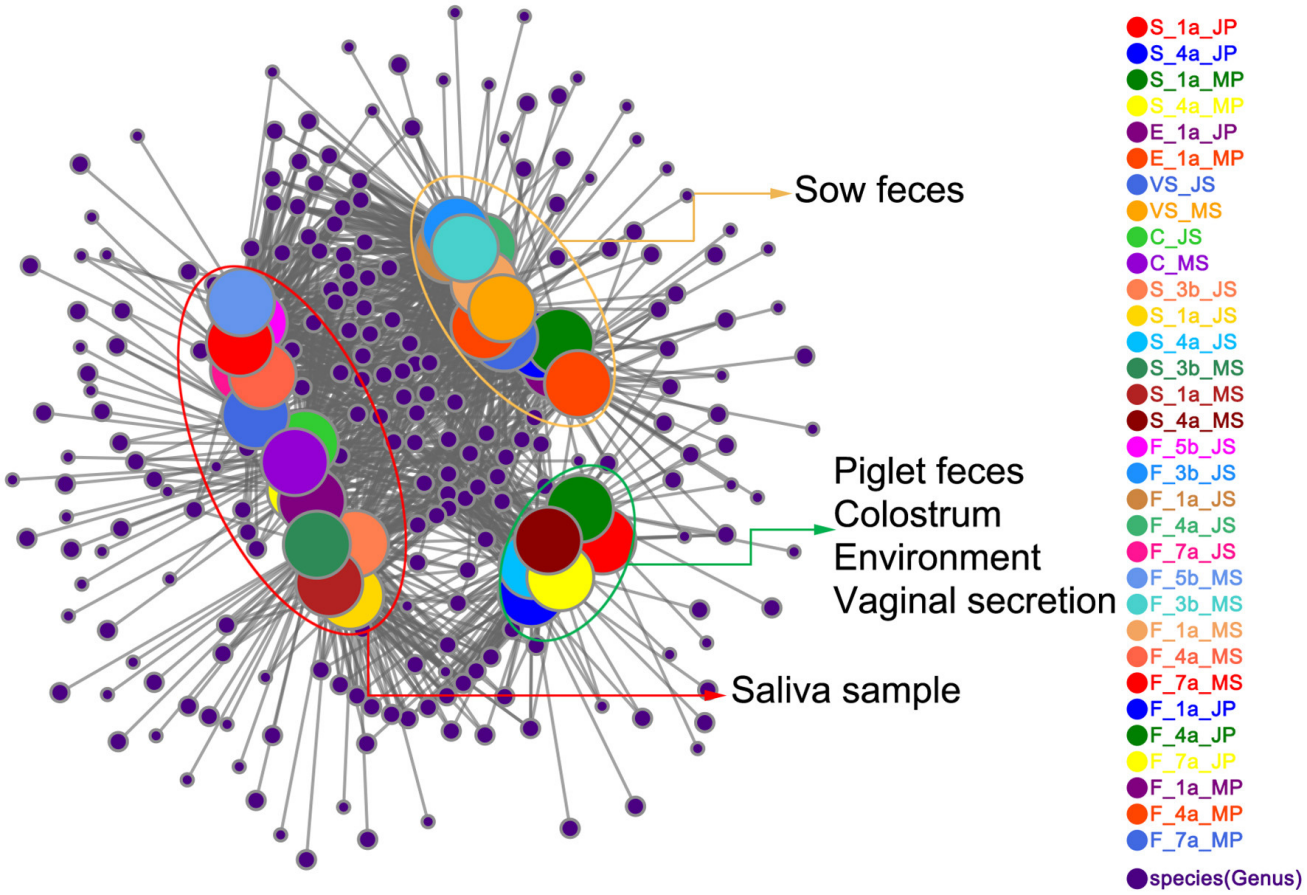
Collinearity network analysis of Jinfen White pig and Mashen pig. Collinear relationships among microbiota of sow and piglet feces, sow and piglet saliva, farrowing bed environment, colostrum, and sow vaginal secretion. The node in the network represents the species node, and the connection between the species node represents that the species is included in the sample. Species with abundance >50 are displayed by default.

To visually show the collinear relationships among piglet, maternal, and environmental microbiota, Networkx software was used to construct a collinearity network graph based on OTUs with an abundance >50. All samples from both varieties shared 822 genera; each shared genus was scored according to connectivity (weighted degree), and it was found that among the top 20 genera with an abundance score >10,000 points (Supplementary Table 4 and Figure 3), core genera such as *Pseudomonas, Bacteroides, Clostridium_sensu_stricto_1* were at the center of the network. In addition, fecal samples from the two sow breeds clustered together, as did saliva samples. However, a more peculiar observation was that of piglet fecal samples from the two breeds at 1 and 4 days of age clustering closely with the farrowing bed environment, colostrum, and vaginal secretions, indicating closer relationships with each other. Thus, the collinearity network graph is consistent with PCoA results, further showing that piglet fecal samples at 1 and 4 d were more similar to those of the farrowing bed environment, colostrum, and vaginal secretions; therefore, it was speculated that early microbial colonization in piglets might be influenced more by the farrowing bed environment, colostrum, and sow vaginal secretions.

### Microbial Composition at Different Taxonomic Levels

**Figure 4:**
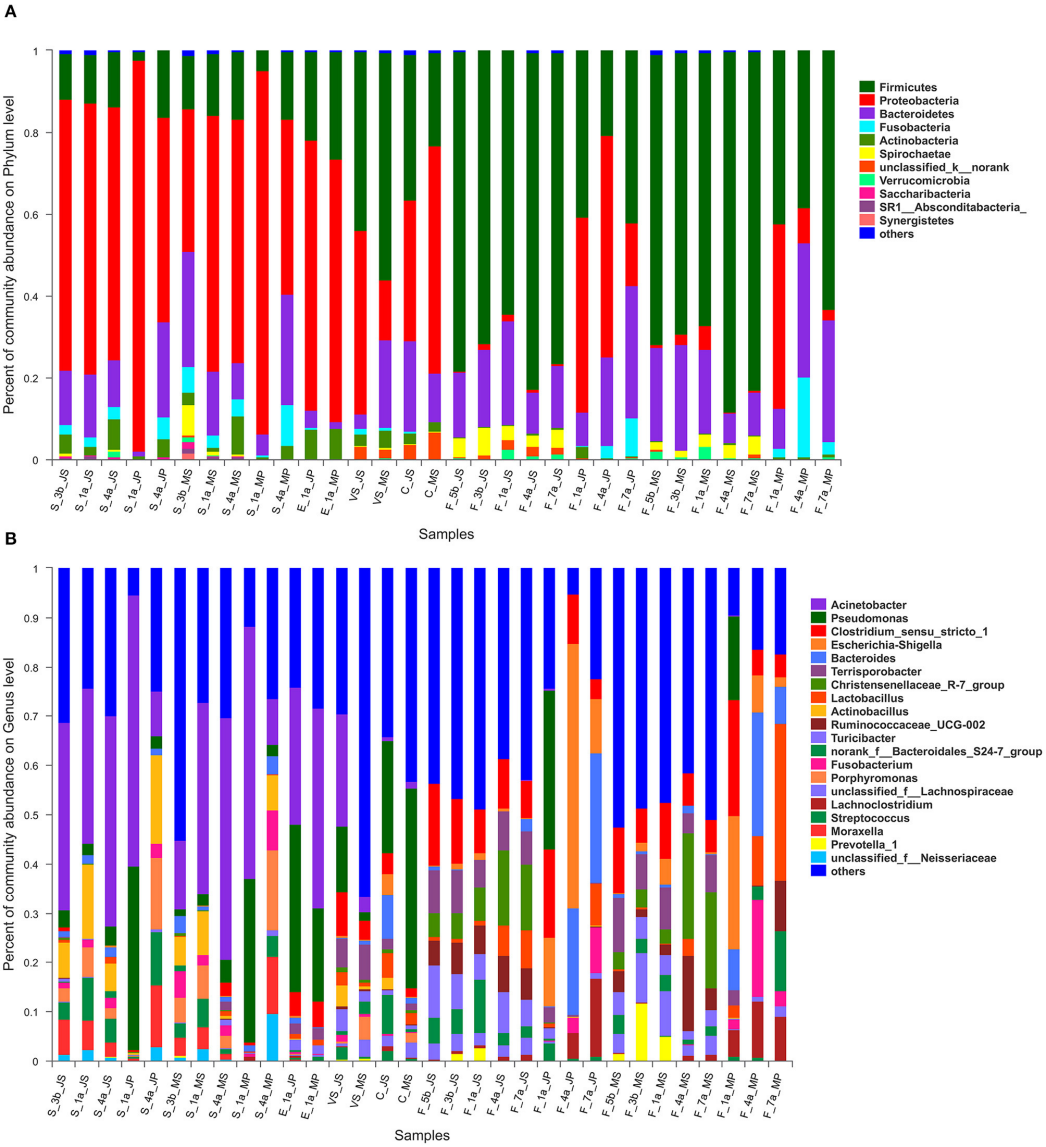
The microbiota composition of Mashen pig and Jinfen White pig. **(A)** The microbiota composition of Mashen pig and Jinfen White pig at the phylum level. **(B)** The microbiota composition of Mashen pig and Jinfen White pig at genera level.

At the phylum level, a total of 11 major phyla with relative abundances >0.01%, including Proteobacteria, Firmicutes, Bacteroidetes, Fusobacteria, and Actinobacteria, were identified in the samples from the two breeds (Supplementary Table 5 and Figure 4A). Proteobacteria was the core phylum in the saliva samples of the two breeds, especially on day 1 in piglets, accounting for 95.4 and 88.9% in Jinfen White and Mashen pigs, respectively—with an increase in piglet age, Proteobacteria abundance decreased gradually, and that of Bacteroidetes began to increase. Microbial composition was similar in vaginal secretions and colostrum samples, with Proteobacteria and Firmicutes being the core phyla in both samples. In addition, the abundance of Firmicutes in sow feces of both breeds gradually decreased before parturition, with an approximate 10% decrease in Firmicutes at parturition, returning to prepartum levels after parturition. In the fecal microbiota of piglets, Proteobacteria first increased and then decreased, while Bacteroidetes increased gradually.

At the genus level, *Acinetobacter* in sow saliva samples before and after parturition was stable at 30–40%; the proportion of *Actinobacillus* in sows before parturition was 7.3%, increasing to 15.1% during parturition, and decreasing by approximately 10% after parturition (Supplementary Table 6 and Figure 4B). *Acinetobacter* and *Porphyromonas* were the predominant genera in piglet saliva samples on day 1, accounting for ~50 and 30%, respectively. *Acinetobacter* began decreasing as piglets grew to the 4th day, while *Actinobacillus, Porphyromonas*, and *Streptococci* increased. The predominant microbiota genera in the environmental samples collected from the farrowing bed were *Acinetobacter* and *Pseudomonas*. The microbiota composition of the two breeds structurally differed, with the proportion of *Acinetobacter* being 27.7 and 40.64% in the environmental microbiota of the Jinfen White and Mashen pigs, respectively. In contrast, the abundance of *Pseudomonas* was ~2-fold greater in the Jinfen White pigs than in the Mashen pigs. The abundance of *Acinetobacter, Pseudomonas*, and *Clostridium_sensu_stricto_1* was higher in vaginal secretion samples from Jinfen White sows than in those from Mashen sows. The core genus in the colostrum samples was *Pseudomonas*, the abundance of this genus in the colostrum samples of Jinfen white pigs was only 1/2 of that of Mashen pigs, and the ratio of *Pseudomonas* in the two breeds was 22.8 and 40.5%, respectively. In the fecal samples, the predominant genera in sows before and after parturition were *Clostridium_sensu_stricto_1, Christensenellaceae_R-7_group, Ruminococcaceae_UCG-002*, among others. The abundance of *Christensenellaceae_R-7_group* increased at the time of parturition, and that of *Ruminococcaceae_UCG-002* was not affected by parturition, remaining stable at ~5%. In addition, the abundance of *Lactobacillus* gradually increased.

### Difference Analysis of Microbiota in Different Sample Types

Combining PCoA, collinearity network, and compositional analyses at different taxonomic levels, we speculated that the early microbial colonization of piglets might be greatly influenced by the farrowing bed environment, colostrum, and vagina secretions. To prove the above inference, the Kruskal–Wallis rank-sum test for the two breed samples (sow vs. piglet saliva; sow colostrum, vaginal secretions, and the farrowing bed environment vs. piglet feces at 1 d old, and sow vs. piglet feces) were conducted at the genus level, with a 95% confidence interval, to analyze differences in genera between samples, and provide an in-depth dissection of factors influencing microbial colonization in newborn piglets.

The abundance of *Acinetobacter, Actinobacillus, Porphyromonas*, and *Streptococcus* was higher in the saliva samples (Supplementary Figure 1). Of these, *Acinetobacter* was most abundant, changing little before and after parturition in sows and after birth in piglets, but not reaching a significant level (*P* > 0.05). The abundance of *Pseudomonas* was the highest in the saliva samples of piglets on the 1st day in both breeds, and its abundance was significantly higher in piglet saliva than in that of sows (*P* < 0.05). The abundance of *Actinobacillus* and *Porphyromonas* increased at first and then decreased form 3 days before parturition to 4 days after parturition, reaching the highest at the time of parturition in sows and piglets on the 1st day (*P* < 0.01). *Moraxella* was most abundant in piglets on the 4 d of age, and the abundance in saliva of piglets was significantly higher than that of sows (*P* < 0.05). Generally speaking, the abundance of *Actinobacillus* in saliva of Jinfen White pigs was higher than that of Mashen pigs, while the abundance of *Porphyromonas* and *Moraxella* was similar between the two breeds.

Compared with the fecal microbiota of piglets, *Pseudomonas* was the only genus more abundant in colostrum, vaginal secretions, and the farrowing bed environment (Supplementary Figure 2). The abundance of *Pseudomonas* in fecal samples of both piglet breeds on the 1st day of birth was closer to that in the farrowing bed environment. *Acinetobacter* was more abundant in the farrowing bed environment of the two breeds, followed by vaginal secretions of Jinfen White pigs. *Clostridium_sensu_stricto_1* and *Escherichia-Shigella*, the core genera in fecal microbiota, were generally not highly abundant in the other samples. The abundance of *unclassified_k__norank* in colostrum was significantly higher than that in piglet feces (*P* < 0.05).

*Clostridium*_*sensu*_*stricto*_*1* was identified in the two sow breeds before and after parturition and in piglet feces, but there was no significant difference between sows and piglets (*P* > 0.05) (Supplementary Figure 3). However, the abundance of *Escherichia-Shigella* was higher in piglet feces; it was found in the feces of Jinfen White piglets on the 1st day following parturition, reaching a high at 4 d of age that was significantly higher than that for other days (*P* < 0.01). The highest abundance of *Escherichia-Shigella* was found in the feces of Mashen piglets on the 1st day after parturition and then began declining gradually. In the fecal samples, the abundance of *Christensenellaceae_R-7_group* in the two sow breeds was significantly higher at 4 and 7 d of age than that on other days (*P* < 0.01). *Bacteroides* were present at a higher abundance in Jinfen White piglets at 4 and 7 d of age, and Mashen piglets at 4 d of age, which was significantly higher than that at other days of age (*P* < 0.05). Another genus, *Terrisporobacter*, was present at a significantly higher abundance in sow feces relative to piglet feces (*P* < 0.01).

### Heatmap Analysis of Microbiota in Different Sample Types

**Figure 5:**
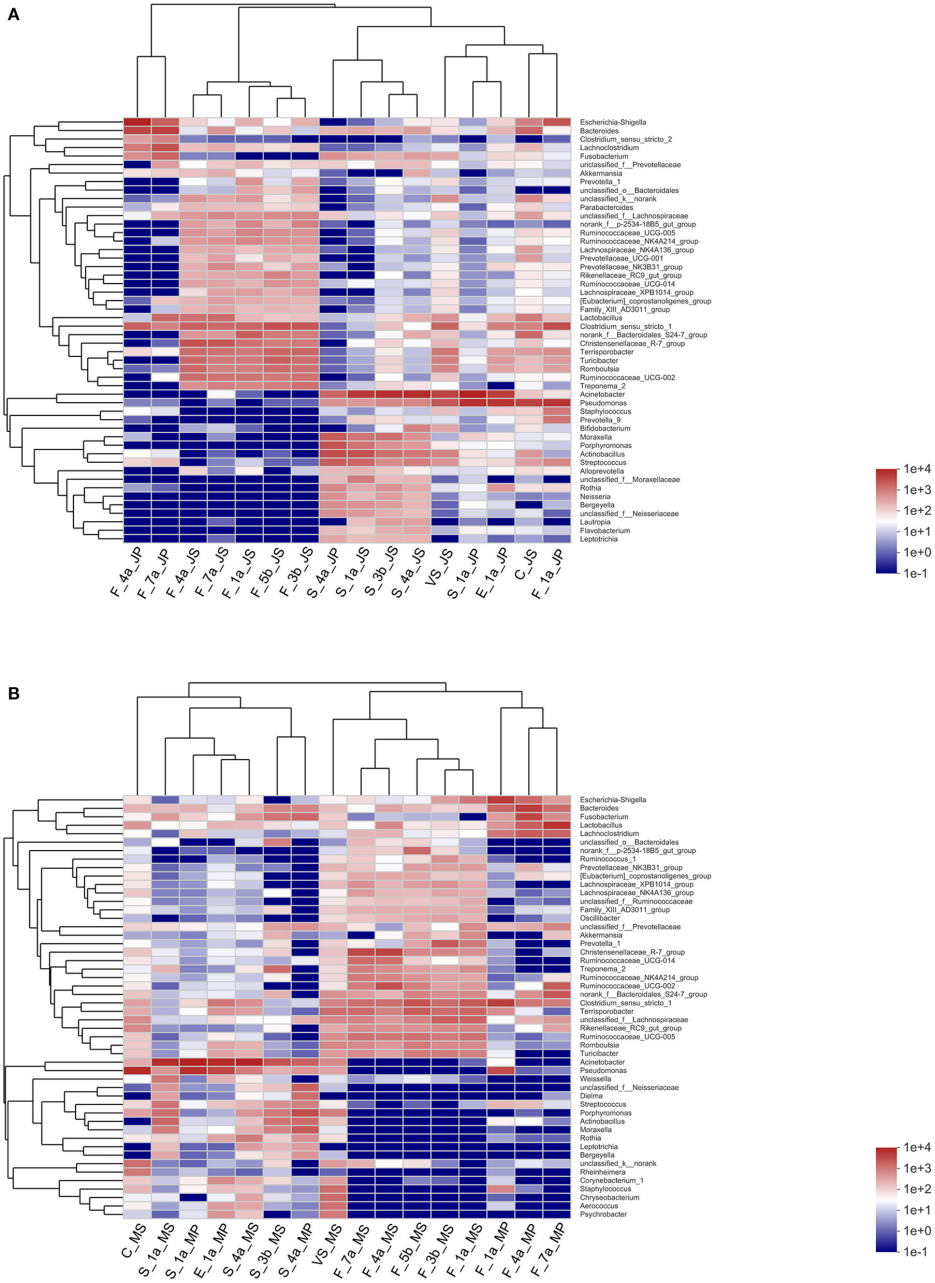
Hierarchical clustering heatmap of the piglets and maternal factors and postpartum environmental samples based on association with microbiota. **(A)** Heatmap analysis results of correlation of Jinfen White pig. **(B)** Heatmap analysis results of correlation of Mashen pig. Columns correspond to the piglets and maternal factors and postpartum environmental samples; rows correspond to the top 50 abundant microbiota based on Spearman's rank correlation coefficient. Red and blue denote positive and negative associations, respectively.

Heatmap analysis was performed using hierarchical clustering of the top 50 abundant microbiota based on Spearman's rank correlation coefficient, providing a greater understanding of the relationship between Jinfen White piglets and maternal factors and environmental samples (Supplementary Table 7 and Figure 5A). The fecal microbiota composition for piglets at 4 and 7 d of age was similar, with higher abundances of *Escherichia-Shigella* and *Bacteroides* in these 2 days. The microbiota in sow feces did not change much before and after parturition, and the fecal microbiota composition for piglets at 4 and 7 d of age was more similar to that of sows. In the saliva samples, *Acinetobacter* had a higher abundance in sows and piglets; the saliva samples of piglets at 4 d were similar to those of sows, while the microbial structure in saliva samples of piglets at day 1 was closer to that of vaginal secretions, environment, colostrum, and feces of piglets on the 1st day following parturition. Among these samples, the microbiota in piglet feces at 1 d of age was more similar to those in colostrum and the environment. Therefore, it is suggested that the intestinal microbial colonization of Jinfen White piglets was more influenced by the colostrum and farrowing bed environment.

The abundance of *Pseudomonas, Acinetobacter*, and *unclassified*_*f* __*Neisseriaceae* and *Dielma* was higher in Mashen pig colostrum, saliva microbiota of sows at parturition and piglets at 1 d of age, and sow saliva, respectively. However, *unclassified*_*f* __*Neisseriaceae* and *Dielma* abundance in the saliva microbiota of piglets at 1 d of age was much lower than that of sows (Supplementary Table 8 and Figure 5B). The microbial composition in the environmental samples was similar to that in piglet saliva, and all the microbiota in colostrum, sow saliva, and environmental samples considerably differed from the fecal microbiota composition of piglets at 1 d of age, which led to the speculation that colostrum in Mashen pigs and the environment had little effect on early microbial colonization in piglets. In the fecal samples where the microbial composition in sows before and after parturition was similar to that of piglets, the predominant core genera were *Escherichia-Shigella* and *Bacteroides*, and there were no large differences in the composition between vaginal secretions and feces. Therefore, it is tempting to speculate that early microbial colonization in Mashen piglets might be more influenced by microbiota in sow feces and vaginal secretions.

Through the heatmap analysis combined with the collinearity analysis and microbial composition, *Lactobacillus, Pseudomonas, Escherichia-Shigella, Bacteroides*, and *Clostridium_sensu_stricto_1* were found to be core genera in the intestinal microbiota of newborn piglets. Colostrum, vaginal secretions, and saliva were the primary sources of intestinal microbiota in newborn piglets, in which *Bacteroides* came from vaginal secretions, *Lactobacillus* from colostrum, *Escherichia-Shigella, Pseudomonas*, and *Clostridium_sensu_stricto_1* came from saliva. *Prevotella 9* was found in higher abundance in sow colostrum but not in early piglet microbiota. The structure of the intestinal microbiota of adult pigs significantly differed from that of piglets, with *Terrisporobacter* being found independently in the vaginal secretions and fecal microbiota of pregnant sows. The microbiota in the saliva of sows and piglets was similar in structure to the environmental microbiota, with a predominance of genera such as *Proteobacteria, Acinetobacter, Actinomycetes*, and *Pseudomonas*.

## Discussion

It has been well-established that intestinal microbiota is an integral part of the body, and the intestine and its microbiota play a vital role in animal growth and immunity. In particular, early intestinal microbial colonization is essential for establishing a stable and healthy microecological structure; thus, it is important to explore the factors that affect intestinal microbial colonization and diversity structure changes in piglets (40, 41). Multiple factors influence the intestinal microbiota of piglets during early colonization; therefore, the diversity of microbiota from different sources such as the dam and piglet environment before and after birth were analyzed. Furthermore, the relationship between early intestinal microbiota and maternal factors and piglet environment was discussed to clarify the rules and factors affecting intestinal microbial colonization of piglets.

Through the study on the intestinal microbial diversity of piglets, it was found that Firmicutes, Bacteroidetes, and Proteobacteria were the core phyla in feces, colostrum, saliva, and vaginal secretions, and the core phylum in saliva, the bed environment, and colostrum was Proteobacteria, and that in feces was Firmicutes. The core microbiota at the genus level in feces has been observed to be *Clostridium sensu stricto, Acinetobacter, Pseudomonas, Escherichia-Shigella*, and *Bacteroidetes* (13). Similar to Chen et al. (13) the core microbiota identified in the present study were *Firmicutes, Bacteroides*, and *Proteobacteria*, with a high abundance of *Acinetobacter, Pseudomonas*, and *Bacteroides*, while *Clostridium_sensu_stricto_1* was highly abundant only at birth and then decreased significantly. Studies have found that *Escherichia-Shigella* is a potential pathogen that may cause dysentery and diarrhea, and breastfeeding may cause more lactic acid production in the intestines of infants, thereby inhibiting and limiting *Escherichia-Shigella* growth (42, 43). Accordingly, in the present study, it was found that the abundance of *Escherichia-Shigella* in the feces of piglets was relatively high in the early stage and then decreased significantly.

In human studies, it has been reported that there is little similarity between the oral microbial community of the mother and infant by analyzing the mode of delivery and microbial diversity of saliva and the nasal cavity, which has no substantial effect on infant early microbial colonization (44). Our study showed a high correlation between microbiota in sow and piglet saliva, which may be due to the pig feeding habit, where sows and piglets feed off farrowing beds and guardrails, promoting microbiota similarity in saliva. Previous studies have demonstrated that the microbiota obtained from vaginally delivered infants is similar to the vaginal microbiota of the mother, which mainly consists of *Lactobacillus* and *Prevotella* (45). Similarly, studies on the microbial similarity between sows and piglets have shown that piglets obtain microbiota not only from sow feces but also from the birth canal, milk, and the environment around the dam; therefore, in addition to fecal microbial transfer, the composition of microbiota in colostrum may also affect the intestinal microbial structure of offspring (46, 47). Furthermore, we revealed that the vaginal secretions of sows, sow colostrum, and piglet feces overlapped, indicating that vaginal secretion and sow colostrum had the greatest influence on the fecal microbiota of piglets and might be the primary source of piglet intestinal microbiota.

To provide a clearer and more direct view of the relationship between microbiota and microbial colonization of piglets across different sample types, community correlation Heatmap analysis was performed. The results indicated that the microbial composition of Jinfen White piglet feces was more similar to that of colostrum and their environment, which were strongly correlated. Thus, it was speculated that the early microbial colonization of Jinfen White pigs was greatly influenced by colostrum and bed environment. On the other hand, the fecal microbiota of Mashen piglets was most similar to that of sows, and it was speculated that early microbial colonization in Mashen pigs was greatly affected by the sow fecal microbiota.

A previous report showed that *Streptococcus, Bifidobacterium*, and *Lactobacillus* in breast milk could be transferred directly to infant intestines; these potentially symbiotic probiotics may help establish early intestinal barriers, furthermore, the phyla Proteobacteria and Firmicutes and the genera *Pseudomonas, Staphylococcus* and *Streptococcus* were the predominant bacterial groups (48); the results of our study show that the core microbiota in colostrum is *Pseudomonas*, which is consistent with the previous study.

In summary, this study utilized 16SrRNA gene sequencing to analyze the variation in microbial diversity characteristics in the feces, saliva, colostrum, and vaginal secretions, as well as the farrowing bed environment of Jinfen White and Mashen sows and piglets. The microbiota in sow feces, colostrum, and vaginal secretions could easily colonize the intestines of piglets through a symbiotic effect. Moreover, the environment and saliva microbiota could also affect the colonization and succession of early intestinal piglet microbiota to some extent—the main colonizing bacteria included *Lactobacillus, Pseudomonas, Escherichia-Shigella, Bacteroides*, and *Clostridium_sensu_stricto_1*. Our results provide a theoretical basis for sow delivery protection and early nursing of piglets, as well as research and development of microbial agents to improve the intestinal health of piglets, and we can further explore the effect of intestinal microbial colonization on the growth and development of piglets in the future.

## Data Availability Statement

The datasets presented in this study can be found in online repositories. The names of the repository/repositories and accession number(s) can be found in the article/Supplementary Material.

## Ethics Statement

The animal study was reviewed and approved by Animal Care and Use Committee of Shanxi Agricultural University.

## Author Contributions

PG, XGu, GC, and BL designed this study. YaL, YM, XGe, CC, and XZ took the samples of Mashen and Jinfen White pigs. YaL and XZ prepared the DNA samples. CL, GL, and YY performed the data analysis. YoL, PG, XGu, and CL wrote the manuscript. All authors contributed to the article and approved the final version.

## Funding

This work was funded by the Key Research Projects in Agriculture of the Shanxi Province (Grant No. 201803D2210221), the University Science and Technology Innovation Project of the Shanxi Province (2021L158), the Program for Sanjin Scholar (Grant Nos. 2016 and 2017), the Fund for Shanxi 1331 Project (Grant Nos. 2017 and 2020), and the National Natural Science Foundation of China (Grant No. 31872336).

## Conflict of Interest

XG was employed by Inner Mongolia Mengniu Dairy Company Limited. XZ was employed by Allwegene Technologies Incorporation. The remaining authors declare that the research was conducted in the absence of any commercial or financial relationships that could be construed as a potential conflict of interest.

## Publisher's Note

All claims expressed in this article are solely those of the authors and do not necessarily represent those of their affiliated organizations, or those of the publisher, the editors and the reviewers. Any product that may be evaluated in this article, or claim that may be made by its manufacturer, is not guaranteed or endorsed by the publisher.
