# Supplementary material for: Effects of Maternal Factors and Postpartum Environment on Early Colonization of Intestinal Microbiota in Piglets
Source: Front Vet Sci. 2022 Apr 7;9:815944. doi: 10.3389/fvets.2022.815944 (PMC9021831; doi:10.3389/fvets.2022.815944)
Supplement: Supplementary file 10 [file Data_Sheet_2.PDF]

Supplementary Figure 2

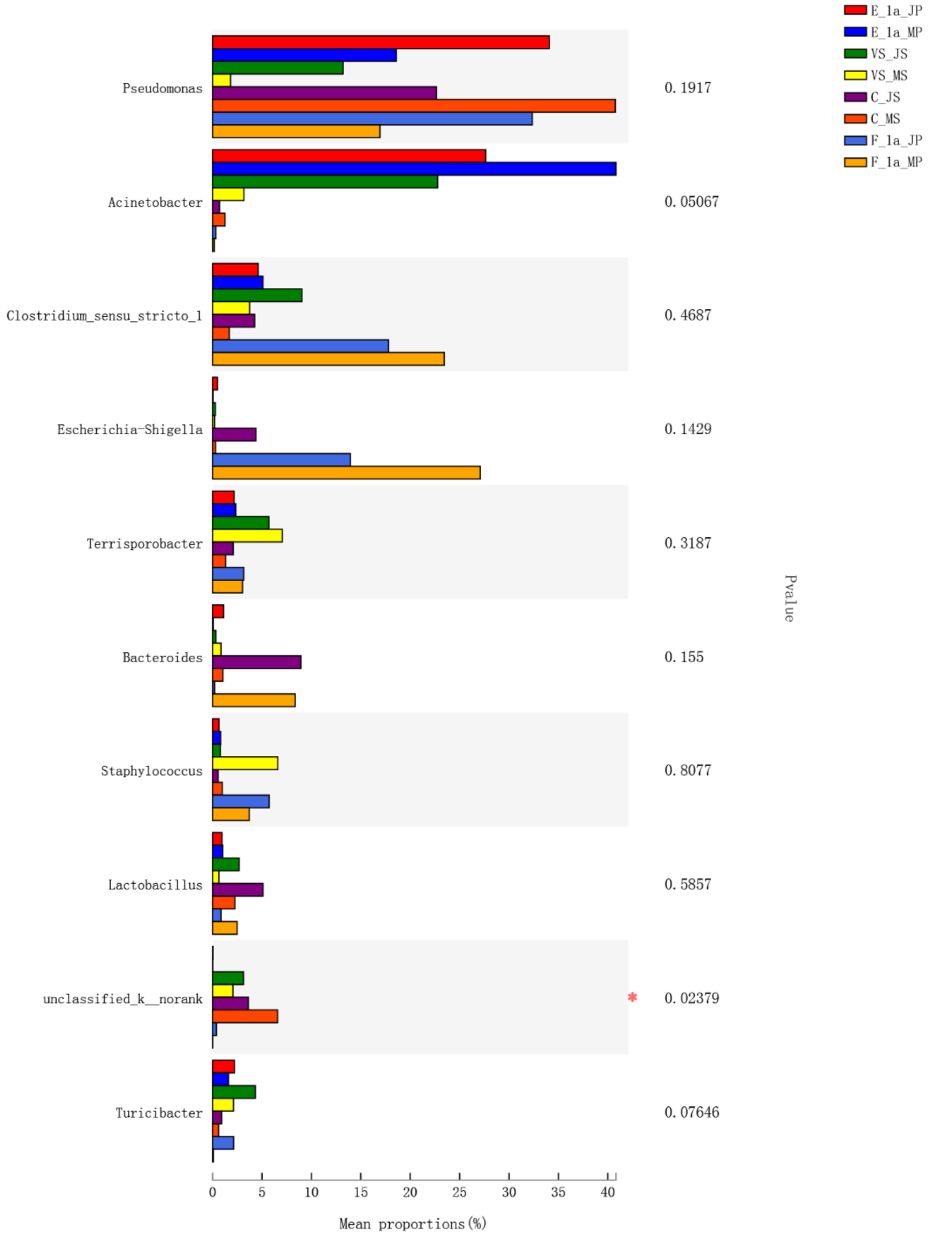

Supplementary Figure 2. Difference analysis of the colostrum and vaginal secretions of Mashen pig and Jinfen White pig and the farrowing bed environment and fecal samples of newborn piglets.

The Y axis represents the microbiota at the genus level, the X axis represents the average relative abundance in different groups, and the columns of different colors represent different groups. On the far right is the P value, * $0.01 < P \leq 0.05$, ** $0.001 < P \leq 0.01$, *** $P \leq 0.001$.
