# Supplementary material for: Effects of Maternal Factors and Postpartum Environment on Early Colonization of Intestinal Microbiota in Piglets
Source: Front Vet Sci. 2022 Apr 7;9:815944. doi: 10.3389/fvets.2022.815944 (PMC9021831; doi:10.3389/fvets.2022.815944)
Supplement: Supplementary file 11 [file Data_Sheet_3.PDF]

Supplementary Figure 3

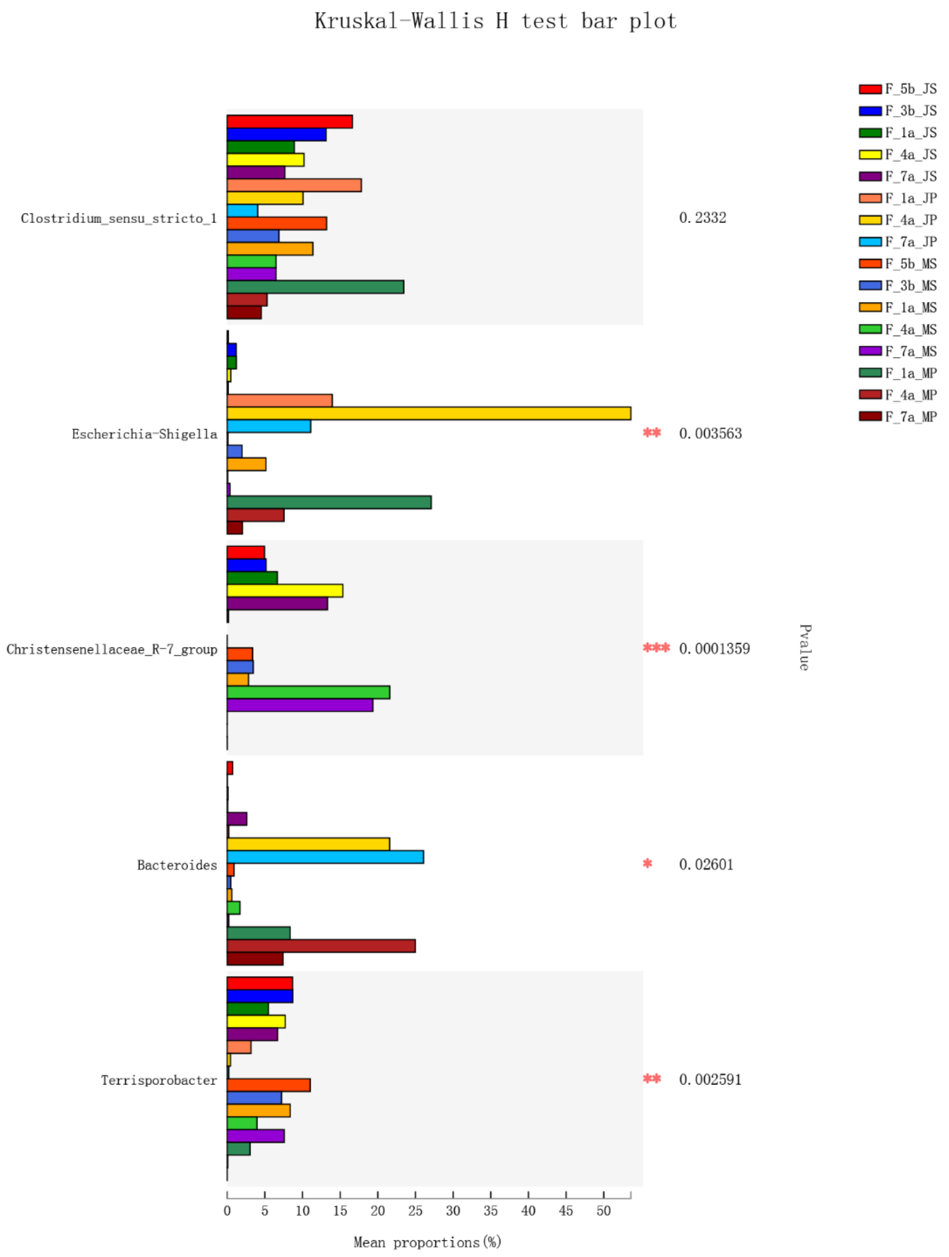

Supplementary Figure 3. Difference analysis of fecal samples of Mashen pig and Jinfen White pig.

The Y axis represents the microbiota at the genus level, the X axis represents the average relative abundance in different groups, and the columns of different colors represent different groups. On the far right is the P value, * 0.01 < P ≤ 0.05， ** 0.001 < P ≤ 0.01， *** P ≤ 0.001.
